# Comparison of serum PCT and CRP levels in patients infected by different pathogenic microorganisms: a systematic review and meta-analysis

**DOI:** 10.1590/1414-431X20176783

**Published:** 2018-05-28

**Authors:** Jun-Hua Tang, Dong-Ping Gao, Peng-Fei Zou

**Affiliations:** 1 Department of Respiration, The First People’s Hospital of Fuyang Hangzhou, Hangzhou, China; 2 Department of Pharmacy, Hangzhou Cancer Hospital, Hangzhou, China; 3 Department of Infectious Disease, Zhejiang University International Hospital, Hangzhou, China; 4 Department of Infectious Disease, Shulan (Hangzhou) Hospital, Hangzhou, China

**Keywords:** Pulmonary infection, PCT, CRP, Bacteria, Fungus

## Abstract

To avoid the abuse and misuse of antibiotics, procalcitonin (PCT) and C-reactive protein (CRP) have been used as new approaches to identify different types of infection. Multiple databases were adopted to search relevant studies, and the articles that satisfied the inclusion criteria were included. Meta-analyses were conducted with Review Manager 5.0, and to estimate the quality of each article, risk of bias was assessed. Eight articles satisfied the inclusion criteria. The concentrations of both PCT and CRP in patients with bacterial infection were higher than those with non-bacterial infection. Both PCT and CRP levels in patients with G− bacterial infection were higher than in those with G+ bacterial infection and fungus infection. In the G+ bacterial infection group, a higher concentration of CRP was observed compared with fungus infection group, while the difference of PCT between G+ bacterial infection and fungus infection was not significant. Our study suggested that both PCT and CRP are helpful to a certain extent in detecting pneumonia caused by different types of infection.

## Introduction

Pulmonary infection is commonly treated by antibiotic therapy in primary care, and has high morbidity and mortality (1,2). Excessive use of antibiotics is the main cause of increased antibiotic resistance. It has been reported that inadequate antimicrobial treatment affects morbidity and mortality (3–5). Therefore, an appropriate disease assessment is a vital early step in the judicious use of antibiotics and management of patients (6,7). Moreover, a sensitive and specific marker that could recognize bacterial infections early is needed.

The identification of pulmonary infection in adults should be conducted to enable appropriate investigation and prompt treatment (8,9). Several methods have been applied to detect pulmonary infection, including clinical symptomatology, radiological examination, inflammatory markers, blood culture, cytology, and microbiology (1). Serum biomarkers such as white cell count, lactate dehydrogenase, leukocyte count, and glucose have also been shown as effective detection methods (10). Ideal indices require accurate identification of infectious and non-infectious disease, and easy and rapid application and detection. As clinical signs and symptoms of infection and laboratory parameters are often inconclusive and some serum biomarkers are elevated in non-infective inflammatory processes, procalcitonin (PCT) and C-reactive protein (CRP) have been studied as novel biomarkers in infectious and inflammatory diseases (11).

PCT and CRP are new approaches used to guide antibiotic therapy and have been researched as markers of infection in serum and pleural fluid. Normally, the concentration of serum PCT is negligible or relatively low with a viral infection, and after a bacterial infection, the levels increase significantly (12). Previous studies have reported that PCT in pleural fluid have no clinical use in diagnosis or prognosis, while serum PCT may have a role in differentiating pulmonary infection (13,14). Serum levels of PCT are increased with a bacterial infection, while levels are unchanged or only moderately increase in a non-infection condition (5,15). CRP, secreted by the liver in response to bacterial infections, is another parameter used to diagnose infection (15). It is synthesized within 4–6 h after the occurrence of inflammation and could peak after around 36 h (16,17). This study sought to assess the difference of serum PCT and CRP concentrations in patients infected by different microorganisms, including G+ bacteria, G− bacteria, and fungus.

## Material and Methods

### Search strategy

To search the relevant published citations, multiple electronic databases including PubMed, Springer, EMBASE, OVID, and China Full-text Journal Database were used without language restrictions. To maximize the search accuracy, the following MeSH terms were assembled with the Boolean operator “OR”: 1) pulmonary infection OR lung infection OR respiratory infection; 2) procalcitonin OR PCT OR C-reactive protein OR CRP. Related articles with any publication status (published, unpublished, in press, and in progress) published from January 2000 to January 2016 were systematically searched and reviewed. Two authors (J-H Tang and D-P Gao) of our team searched the literature independently and examined the reference lists to obtain additional relevant studies that were not identified.

### Study selection

Two authors (J-H Tang and D-P Gao) selected the citations independently with the following inclusion criteria: 1) adult patients with pulmonary infection; 2) sample size more than 50; 3) a randomized control trial or controlled clinical trial; 4) comparison of PCT or CRP between patients with pulmonary infection and control; and 5) availability of full text. The exclusion criteria were: 1) non-randomized studies; 2) studies on other diseases rather than pulmonary infection; and 3) studies lacking outcome parameters or comparable results. They screened the titles and abstracts of the articles, and subsequently, the full text of the studies that potentially met the criteria was obtained. The two investigators determined the included articles together, and disagreements were resolved by consultation with a third investigator, if necessary.

### Data extraction

After reading the full text of the articles, the characteristics from each study were extracted using a standard data extraction: the first author's name, year of publication, year of onset, age range of patients, gender distribution (male/female), sample size (infection/control), pathogenic microorganism, and parameters. Pathogenic microorganism in this study included bacteria and fungus, and in some articles, bacteria were subdivided into G+ and G−. The parameters included PCT, CRP or both.

### Statistical analysis

Meta-analyses were conduct with the software Review Manager 5.0 (Cochrane Collaboration, 2011) to estimate the serum concentration of PCT and CRP in patients with or without pulmonary infection among selected articles. For continuous outcomes, standard mean difference (SMD) with 95% confidence intervals (CIs) of serum PCT and CRP were calculated. P<0.05 was considered statistically significant. Heterogeneities in this study were assessed using the I^2^ index. We chose the random-effect model when the I^2^ statistic was >50%, otherwise the fixed-effect model was applied.

In addition, the quality of the studies was assessed with sensitivity analysis and bias analysis. Risk of bias was independently assessed according to the Cochrane Handbook for Systematic Reviews of Interventions by two members of our team. In case of disagreement, a third investigator was the adjudicator. To estimate possible publication bias, funnel plot and Egger's test was conducted with STATA 10.0 software.

## Results

### Search results

**Figure 1.**
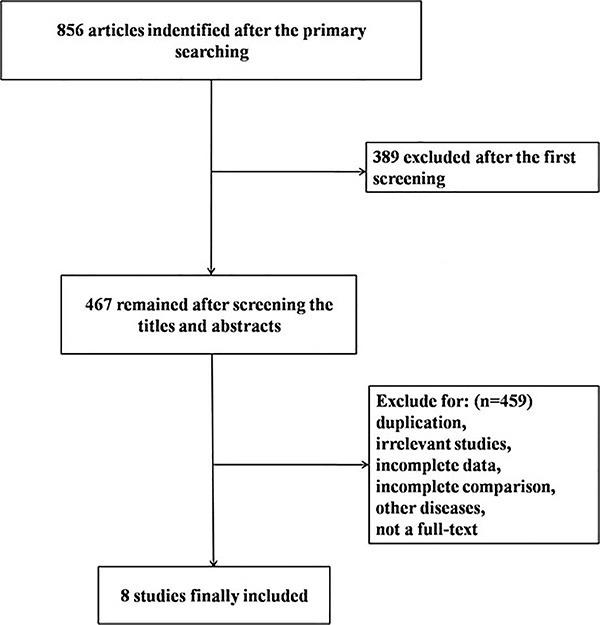
Flow diagram of the study selection process and the reasons for exclusion.

As shown in the flow diagram of Figure 1, 856 relevant studies were initially found, and 848 articles were excluded for duplication, irrelevant studies, incomplete data, incomplete comparison, other diseases, and not a full-text. Finally, 8 articles (18–25) satisfied the inclusion criteria. Among these, 3 studies assessed only bacterial infection, and the other 5 included G+ bacteria, G− bacteria, and fungus infection.

### Characteristics of included studies

Detailed characteristics of the included studies are reported in Table 1. All studies were published from 2011 to 2016. The sample size ranged from 69 to 338. In total, 876 patients with pulmonary infection and 443 without pulmonary infection were included in the analyses.

**Table 1.** Characteristics of the included studies.

Author	Year	Year of onset	Age range	Gender distribution (male/female)	Sample size (infection/control)	Pathogenic microorganism	Parameters
Chen YJ 18	2016	Apr 2012 to Apr 2015	19–79	57/43	100 (50/50)	G+ bacteria, G− bacteria, Fungus	PCT, CRP
Du HS 19	2011	Jan 2013 to Dec 2013	18–82	105/104	210 (131/79)	G+ bacteria, G− bacteria, Fungus	PCT, CRP
Porfyridis 20	2014	Nov 2010 to Jan 2012	Infection: 79.6 ± 15.4; Control: 79.8 ± 6.3	54/33	87 (58/29)	Bacteria	PCT, CRP
Sun WF 21	2011	Dec 2008 to Dec 2010	13–78	36/33	69 (39/30)	Bacteria	PCT, CRP
Wang XD 22	2016	May 2014 to Dec 2015	37–79	207/131	338 (280/58)	G+ bacteria, G− bacteria, Fungus	PCT
Xiao L 23	2015	Jan 2014 to Jan 2015	17–80	86/74	160 (120/40)	G+ bacteria, G− bacteria, Fungus	PCT, CRP
Yang AL 24	2014	Jun 2011 to Aug 2012	38–69	88/68	156 (78/78)	Bacteria	PCT, CRP
Zhang JY 25	2015	Jul 2013 to Aug 2014	19–78	100/100	200 (120/80)	G+ bacteria, G− bacteria, Fungus	PCT, CRP

PCT: procalcitonin; CRP: C-reactive protein.

### Quality assessment

**Figure 2.**
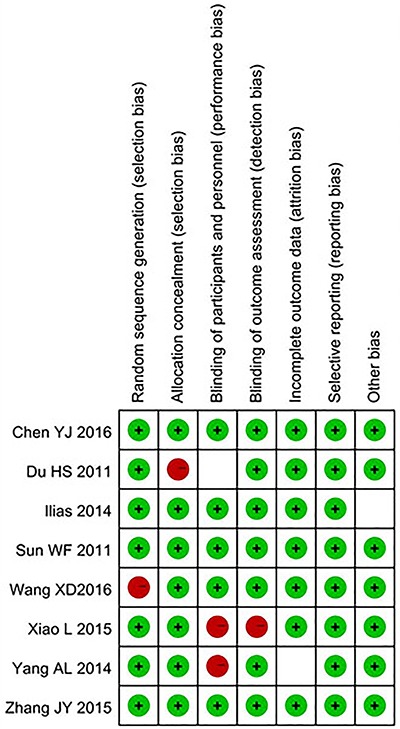
Quality assessment of the included studies. Green: low risk of bias; Blank: unclear risk of bias; Red: high risk of bias.

The results for risk of bias are shown in Figure 2.

### Meta-analysis of the detection indices

**Figure 3.**
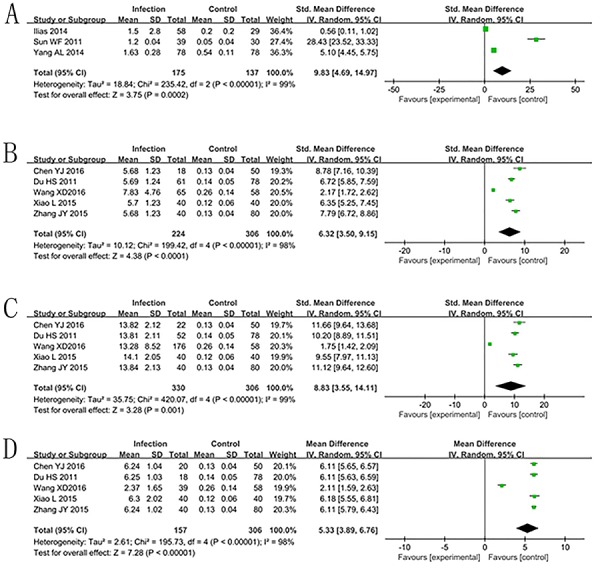
Forest plot for the concentration of procalcitonin (PCT) between *A*) bacterial infection and control group, *B*) G+ bacterial infection and control group, *C*) G− bacterial infection and control group, and *D*) fungus infection and control group.

**Figure 4.**
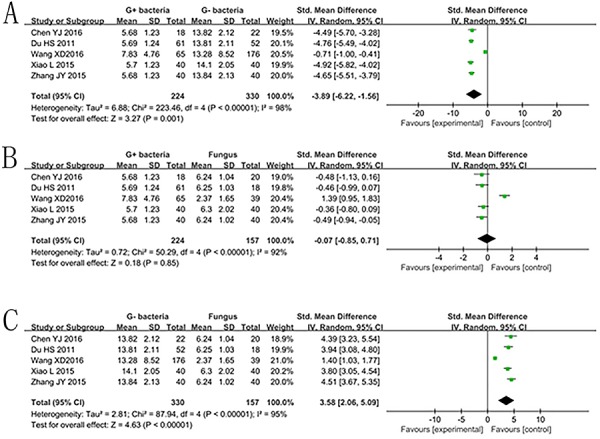
Forest plot for the concentration of procalcitonin (PCT) between *A*) G+ bacterial infection and G− bacterial infection group, *B*) G+ bacterial infection and fungus infection group, and *C*) G− bacterial infection and fungus infection group.

*PCT*. Forest plots for the concentration of PCT between different groups are presented in Figures 3 and 4. The meta-analyses results showed that the concentration of PCT in patients with bacterial infection was much higher than that of control. When bacterial infection was subdivided into G+ and G− bacterial infection, the concentration of PCT of these two group were significantly above the concentration of the control group. Besides, the concentration of PCT in patients with fungus infection exceeded that of the control group.

The concentration of PCT in patients with G− bacterial infection was much higher than that of G+ bacterial infection and fungus infection group, while the difference between G+ bacterial infection and fungus infection was not significant.

**Figure 5.**
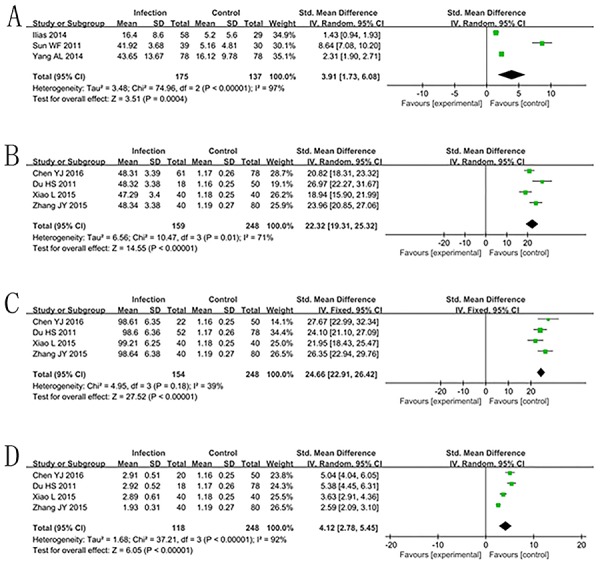
Forest plot for the concentration of CRP between *A*) bacterial infection and control group, *B*) G+ bacterial infection and control group, *C*) G− bacterial infection and control group, and *D*) fungus infection and control group.

**Figure 6.**
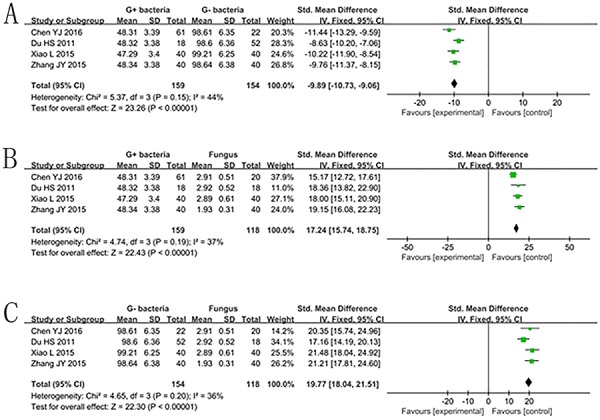
Forest plot for the concentration of CRP between *A*) G+ bacterial infection and G− bacterial infection group, *B*) G+ bacterial infection and fungus infection group, and *C*) G− bacterial infection and fungus infection group.

*CRP*. Figures 5 and 6 show the comparisons of CRP concentrations between different groups. The result suggested that the concentration of CRP in patients with bacterial infection was much higher than that of control. Four of the 8 included studies assessed the concentration of CRP in patients infected by different pathogenic microorganism. All patients with G+ bacterial, G− bacterial, and fungus infections had higher concentration compared with the control group.

The concentration of CRP in patients infected by G− bacteria was much higher than that of G+ bacterial infection and fungus infection groups, and the G+ bacterial infection also had higher concentrations than the fungus infection group.

### Bias analysis

Despite the high heterogeneities of the included studies, we were not concerned about publication bias as only 8 articles were included (26).

## Discussion

The use of PCT and CRP as biomarkers for discriminating bacterial infection has been discussed in various studies, but this is the first meta-analysis that involved comparing the difference of PCT and CRP in patients infected by different pathogens.

Decisions about antibiotic treatment for infections are made by physicians based on detection results (27,28). In recent years, PCT and CRP are the two most common markers, which are easy to assess and have high sensitivity and specificity (29,30). It is known that serum PCT levels are higher in bacterial, fungal, and parasitic infections than in viral infections or non-infected patients, which has made PCT a guide to antibiotic treatment in pneumonia (31,32). The results of this study showed that serum PCT concentration was significantly higher in patients with bacterial pneumonia than patients without pneumonia. The level was the highest in patients infected by G-bacteria, while the concentration in G+ bacterial infection was as high as that in fungus infection. All these results suggested that PCT levels could be useful in discriminating between these conditions, could help physicians’ decisions on using antibiotics or not.

Previous studies have reported that PCT has a better sensitivity than CRP to differentiate bacterial infections from non-bacterial infections, and the reliability of the application of CRP in guiding antibiotic therapy still had problems (33). Thus, PCT seems more accurate than CRP. Although the power of CRP is lower, it could also help discriminate different types of pneumonia infection. The results are promising because CRP was significantly higher in bacterial infection compared with patients without infection. Besides, unlike PCT, the concentration of CRP in G+ bacterial infection, G− bacterial infection, and fungus infection group was different, which means that physicians could identify the infection by measuring CRP levels.

Though the concentrations of PCT and CRP in different infections were different, it is necessary to establish a cut-off value. Unfortunately, the criteria used for thresholds establishment in the included studies were heterogeneous, perhaps due to the different profile of subjects or inclusion and exclusion criteria. Porfyridis et al. (20) reported that serum PCT levels <1.1 ng/mL were considered normal. We could not differentiate G+ bacterial infection from fungus infection using PCT, as the concentrations were similar. From index comprehensive results, we think that 10 ng/mL could be the cut-off value to distinguish G− bacterial infection from G+ bacterial infection or fungus infection. For CRP, if the concentration is <10 mg/L, we could discard bacterial infection (18,19). As the concentration of CRP in G+ bacterial infection is about 48 mg/L and in G− bacterial infection the value is about two times higher, we think 70∼80 could be the cut-off value to distinguish between them.

According to the above results, we suggest that to a certain extent both PCT and CRP are helpful in differentiating different types of infections, and the levels could aid clinicians in identifying those patients who do not need antibiotics as a supplementary means. By reducing the number of less reliable tests such as leukocyte count and white cell count, and consequently the unnecessary use of antibiotics, the cost-effectiveness of detection is also increased.

Although this study suggested that PCT and CRP could be the markers to diagnose pulmonary infection, there are some potential biases and limitations in our study. First, the increase of antibiotic therapy may reduce the levels of PCT and affect the results. In addition, some studies that were included in our meta-analysis enrolled patients with high willingness to participate and interested in improving treatment and physicians with high motivation, which may have caused selection biases. As high heterogeneities were observed in the meta-analyses, we selected random effect models. The reasons for high heterogeneity are complex and we believe that different test technologies and the limited number of included articles may be the main causes. Thus, in-depth and high-quality research is required to reduce heterogeneities and potential biases.

With the abuse of antibiotics for pulmonary infection, the diagnosis needs to be more sensitive and specific to help the decision-making process. We suggest that both PCT and CRP levels may be helpful in diagnosing infections and distinguishing between different pathogens.
